# Effects of deworming medication on anaemia among children aged 6–59 months in sub-Saharan Africa

**DOI:** 10.1186/s13071-021-05123-4

**Published:** 2022-01-12

**Authors:** Andy Bauleni, Fentanesh N. Tiruneh, Tisungane E. Mwenyenkulu, Owen Nkoka, Gowokani C. Chirwa, Steve Gowelo, Michael G. Chipeta, Peter A. M. Ntenda

**Affiliations:** 1 Malaria Alert Centre, Kamuzu University of Health Sciences, Private Bag 360, Chichiri, Blantyre 3, Malawi; 2 Department of Applied Human Nutrition, Faculty of Chemical and Food Engineering, Bahir Dar Institute of Technology, Bahir Dar University, Bahir Dar, Ethiopia; 3 Department of Clinical Sciences, Academy of Medical Sciences, Malawi University of Science and Technology, PO Box 5196, Limbe, Malawi; 4 Institute of Health and Wellbeing, University of Glasgow, Glasgow, UK; 5 Department of Economics, Chancellor College, University of Malawi, P.O. Box 280, Zomba, Malawi; 6 Geospatial Epidemiology, Big Data Institute, University of Oxford, Old Road Campus, Oxford, OX3 7LF UK

**Keywords:** Coverage, Deworming medication, Anaemia, Haemoglobin, Sub-Saharan Africa

## Abstract

**Background:**

Despite the limited knowledge regarding the effects of deworming medication (DM) on nutritional indicators in sub-Saharan Africa (SSA), deworming programmes continue to be implemented in resource-limited countries. Therefore, the current study aimed to examine the effects of DM on anaemia among children aged 6–59 months in SSA.

**Methods:**

The analysis was performed using data obtained from 17 demographic and health surveys (DHSs) conducted in SSA. Children were considered to be anaemic if their haemoglobin (Hb) concentration was less than 11.0 g/dl, adjusting for altitude. To account for both multiple measures at the cluster level and the clustering of children within the same country, generalized linear mixed models were used to analyse the anaemia outcomes in 50,075 children aged 6–59 months.

**Results:**

Overall, anaemia was reported in 61.8% of the children, and their median Hb concentration was 10.5 g/dl (interquartile range 9.4–11.5). The prevalence of anaemia ranged from 34.5% in Rwanda to 81.1% in Mali. Multivariate analyses showed that children who did not receive DM had increased odds of being anaemic (adjusted odds ratio [aOR]: 1.11; 95% confidence interval [CI] 1.07–1.16).

**Conclusions:**

The current study revealed that DM can decrease the risk of anaemia among preschool-age children (pre-SAC) in SSA. Thus, tailored public health programmes aimed at reducing childhood anaemia need to consider deworming. However, longitudinal studies are needed to validate the association that has been reported in this cross-sectional study.

**Graphical Abstract:**

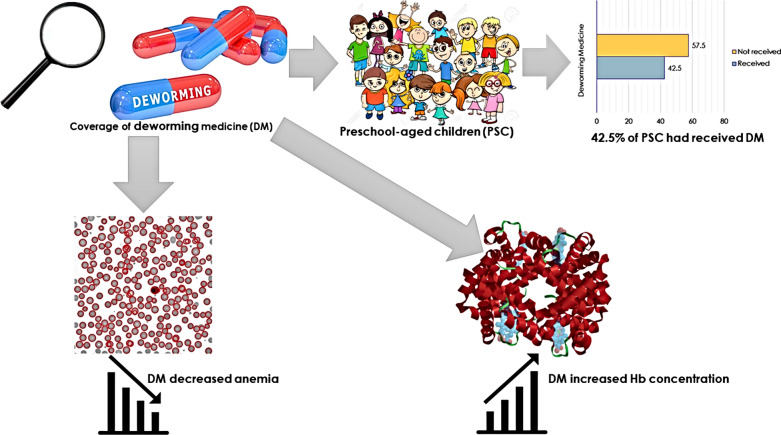

## Background

Globally, soil-transmitted helminth (STH) infections are the most common neglected tropical diseases (NTDs), affecting more than 1.5 billion people, or 24% of the world’s population [1]. Generally, STH infections are broadly distributed in tropical and subtropical parts of the world, with the greatest numbers occurring in sub-Saharan Africa (SSA), the Americas, China, and East Asia. Nonetheless, the highest burden occurs in SSA [1, 2]. STH infections are transmitted from person to person through soil contaminated by human faeces [2]. They affect children living in poverty, usually in an environment that lacks adequate sanitation [3]. The World Health Organization (WHO) reported that approximately 270 million preschool-age children (pre-SAC) reside in settings where these parasites are transmitted [1, 4].

The main species of STHs that affect people include roundworms (*Ascaris lumbricoides*), whipworms (*Trichuris trichiura*), and hookworms (*Necator americanus* and *Ancylostoma duodenale*) [2]. However, all these species are placed under the same group since they share similar diagnostic protocols and quite often respond to the same chemotherapy [1, 2]. Commonly, a single dose of albendazole (400 mg) or mebendazole (500 mg) administered annually or biannually is used for deworming [5]. WHO recommends deworming medication (DM) as a public health strategy for all pre-SAC residing in settings where the baseline prevalence of any STH is 20% or more, in order to reduce the burden of STHs [5, 6].

The WHO 2012 NTD roadmap target aimed at treating at least 75% of pre-SAC where the prevalence in this age group surpassed 20% [7, 8] and 75% coverage with preventive chemotherapy (PC) achieved in pre-SAC in 100% of countries by 2020 [8, 9]. However, because numerous countries were on the verge of achieving this goal, a revised 2020 NTD roadmap with new goals for eliminating STHs as a public health problem by 2030 was proposed [10]. Generally, WHO defines elimination of a public health problem as less than 2% moderate to heavy infections of STHs [11]. Thus, achieving these goals requires both mass distribution of DM and monitoring of prevalence over time. STH infections are a major public health hazard not only because they disturb an individual’s physical development and lead to cognitive and educational deficits, but also because they interfere with nutritional uptake and digestion [3, 12]. Specifically, hookworms may impair the nutritional status of the individuals by feeding on the host tissues, including host blood, which leads to a loss of iron and protein [13]. Furthermore, worms increase malabsorption of nutrients, and may likely compete for vitamin A in the intestine, as well as causing diarrhoea and dysentery [14, 15]. Additionally, STHs, and hookworms in particular, may induce chronic intestinal blood loss that can result in anaemia [1].

Anaemia is a condition in which haemoglobin (Hb) concentration and/or amount of red blood cells (RBCs) are inadequate to meet an individual’s oxygen-carrying capacity [16, 17]. Globally, anaemia is a major global public health hazard that predominantly affects young children [18]. WHO estimates indicate that 42% of pre-SAC worldwide are anaemic [19]. Even though it is well established that iron deficiency is the most important etiology of anaemia, other conditions such as malaria, parasitic infection, nutritional deficiencies, and haemoglobinopathies [21–25] also can all increase the risk of anaemia. Consequently, anaemia may eventually lead to poor developmental growth in children, which has long-term implications such as poor cognitive capacity and poor human capital. Given that human capital is an important input for economic growth, as it provides the necessary labour, anaemia may thus have a direct effect on economic growth and development [20, 21].

Numerous studies have reported the positive association between intestinal parasitic infections and various nutritional outcomes including anaemia among young children [22–25], yet the influence of deworming and its impact on nutritional outcomes have drawn mixed reactions [2, 26–28]. Nonetheless, the implementation of DM and its effects on anaemia among pre-SAC has been inconsistent and problematic. WHO uses the school setting as an ideal platform for public health interventions in many types of health care, including health education, iron supplementation, and DM for parasitic infections [29]. As a result, pre-SAC are less likely to benefit from such programmes, as they are in communities where such programmes are rarely implemented. Accordingly, the principal aim of this study was to examine the effects of DM on anaemia among children aged 6–59 months in SSA. Further, the secondary aim of this study was to examine the coverage and factors associated with DM in pre-SAC. The results of this study will be useful in assessing whether African countries are on track to achieving the African Agenda 2063 aspiration 6 [30] and Sustainable Development Goals (SDGs) 2 and 6 [31], and whether the WHO 2030 elimination goals for STH will be met.

## Methods

### Study design

The current study used secondary data from the 17 demographic and health surveys (DHSs) conducted in SSA. These surveys are comparable and representative (at the national level, residence level, and regional level) cluster-sample household surveys that have been conducted in more than 90 countries globally since 1984 [32]. Details about these surveys can be accessed elsewhere [33].

### Data sources

**Fig. 1:**
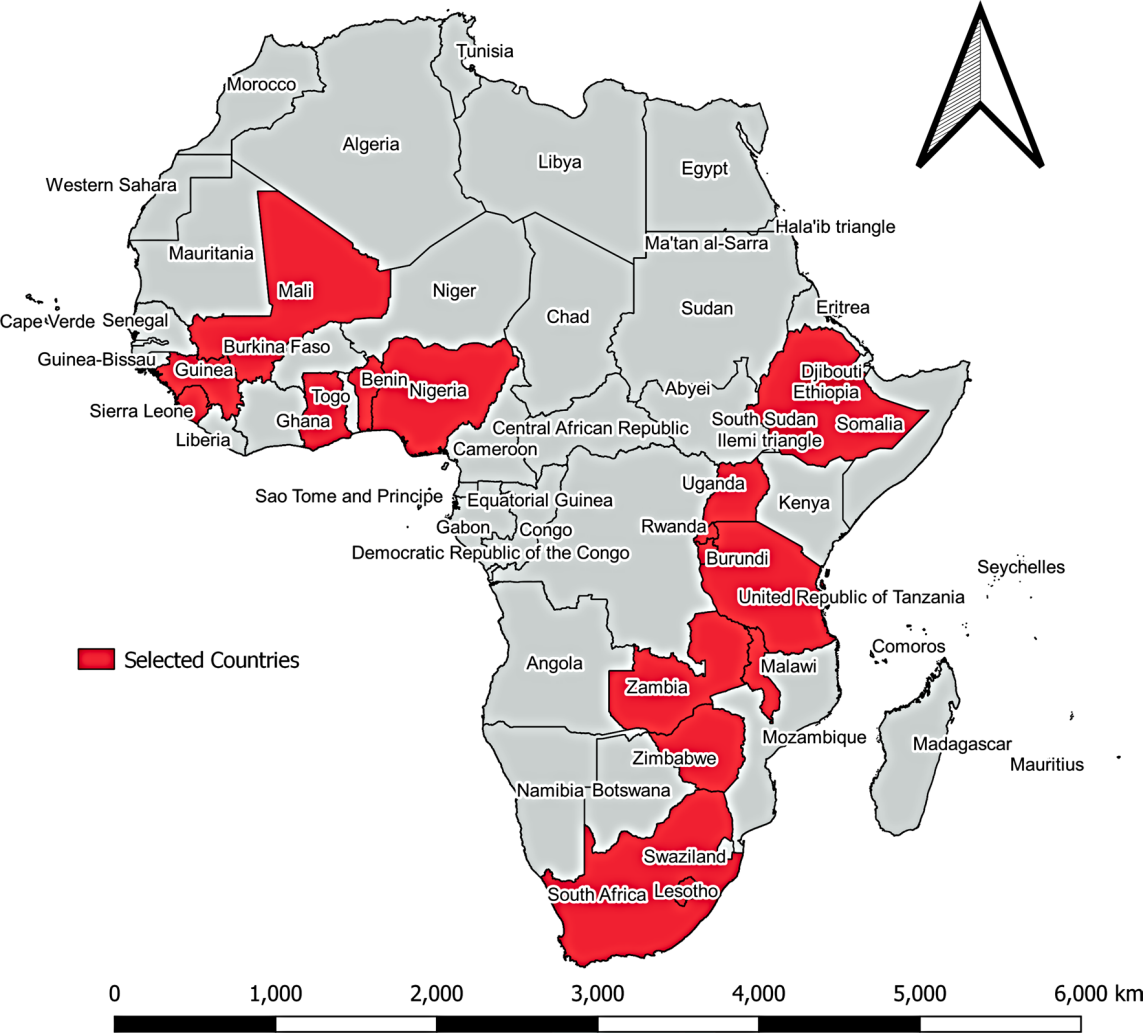
Sub-Saharan African countries selected based on the availability of data (Source: QGIS version 3.16)

We pooled 17 DHSs conducted between 2014 and 2018 in SSA. Specifically, the following countries were included: Benin (2018), Burundi (2018), Cameroon (2018), Ethiopia (2016), Ghana (2017), Guinea (2018), Lesotho (2014), Mali (2018), Malawi (2016), Nigeria (2018), Rwanda (2015), Sierra Leone (2019), South Africa (2016), Tanzania (2016), Uganda (2016), Zambia (2018), and Zimbabwe (2015) [33]. Figure 1 shows the selected SSA countries included in this study. Generally, these countries were selected and included because they had information on both DM and Hb estimates.

### Data collection

In the DHS, information was collected from women aged 15–49 years with under-5 children prior to the survey using the Woman’s Questionnaire. Data on immunization coverage, DM, iron supplementation, vitamin A supplementation, anthropometric measurements and nutritional indicators, recent occurrences of diarrhoea, fever, and cough for young children, and treatment of childhood diseases were collected.

### Inclusion and exclusion

**Fig. 2:**
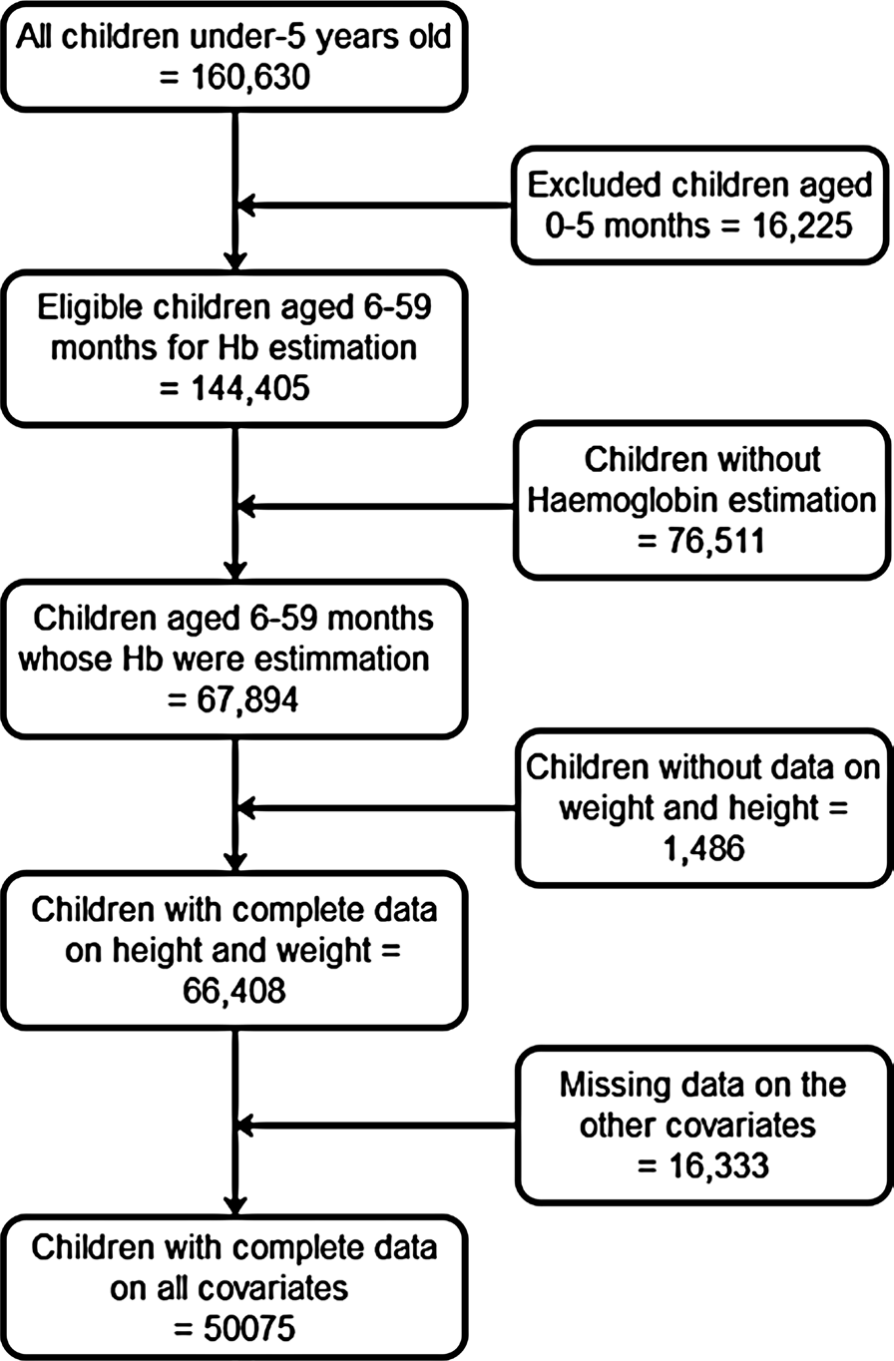
Inclusion and exclusion criteria of the study participants

We focused on pre-SAC because the DHS collects data on that age group and not on school-age children (SAC). We limited our analyses to live children aged 6–59 months as per WHO recommendation. Furthermore, children whose households were not selected for height and weight measurements or who had missing data on the other variables were excluded. This study included children whose caregivers had been interviewed and had provided consent. After applying the inclusion and exclusion criteria, which included age restriction, 50,075 children under 5 years of age were included in the analysis (Fig. 2).

### Anaemia testing

In each country, anaemia testing was performed in the subsample of selected households. Blood samples for anaemia testing were collected from all children aged 6–59 months for whom consent was obtained from their parents or the adult responsible for the children. Blood samples were drawn from a drop of blood taken from a finger prick (or a heel prick from children aged 6–11 months) and collected in a microcuvette. Analysis of Hb was conducted on-site with a battery-operated portable HemoCue analyser.

### Anthropometric measurements

All the DHS surveys included in this study, height, and weight were collected from children under the age of 5 years who resided in the household the previous night prior to the data collection [34]. Weight measurements of the children were collected using electronic SECA 878 flat scales while ShorrBoard^®^ measuring boards were used to record the height measurements. For children less than 24 months old, recumbent height/length measurements were taken, while standing height/length measurements were recorded for children aged 24 months or older [35]. Information on age, height, and weight was used to calculate several childhood nutritional indices (height/length for age, weight for height/length, and weight for age).

### Operationalization of the study variables

#### Outcome variable

The principal outcome variable of this study was anaemia (as measured by Hb). WHO recommendations were used to define anaemia in children aged 6–59 months. Children were considered to be anaemic if their Hb concentration was less than 11.0 g/dl, adjusting for altitude [36].

#### Main explanatory variable

The principal explanatory variable of the current study was DM. Caregivers were asked, “Was (NAME) given any drug for intestinal worms (deworming chemotherapy) in the last 6 months?” The answer to this question was categorized as “yes” if the child had received DM and “no” if the child had not received DM.

#### Potential confounders

Based on insights from relevant literature [37], the following characteristics were treated as potential confounders: child-level factors included sex of the child (male/female), age of the child in months (6–11, 12–23, 24–35, 36–47, and 48–59), presumed fever in the last 2 weeks (no/yes), and an episode of diarrhoea in the last 2 weeks (no/yes). An episode of diarrhoea was defined as the passage of three or more loose or liquid stools in 24 h [38]. We also considered variables that measure the nutritional status of the child, including stunting status (not stunted/stunted) and wasting status (not wasted/wasted). In addition, we considered factors at the maternal and household levels, and these characteristics included the age of the respondents in years (< 25, 25–34, ≥ 35), maternal anaemia (no/yes), education level of the respondents (no formal education, primary education, and secondary and above education), household wealth (poorest, poorer, middle, richer, and richest), amount of media exposure (0, 1, 2, and 3), type of drinking water sources (unimproved/improved), type of household sanitation facility (unimproved/improved), place of residence (urban/rural), and year of the data collection (2014, 2015, 2016, 2017, 2018, and 2019). Childhood stunting and wasting were defined as moderate and severe—that is, below minus two standard deviations (< −2SD) from median height-for-age and weight-for-age *z*-scores of the reference population, respectively [39].

Further, the observations for the household sources of drinking water and type of toilet facility were classified as “improved” and “unimproved” using the revised definitions specified by the WHO/UNICEF Joint Monitoring Programme (JMP) report [40]. Improved water sources included piped water into dwelling, piped water to yard/plot, public tap or standpipe, tube well or borehole, protected dug well, protected spring, and rainwater, while unimproved water sources included unprotected dug well, and unprotected spring, river, dam, lake, pond, stream, canal, and irrigation canal [40]. Improved sanitation facilities included flush toilet, piped sewer system, septic tank, flush/pour flush to pit latrine, ventilated improved pit latrine, pit latrine with slab, and composting toilet. Unimproved sanitation facilities included pit latrines without a slab or platform, hanging latrines or bucket latrines, and open defecation [40].

Maternal anaemia was defined as mothers with Hb levels of less than 12.0 g/dl, adjusting for altitude [36]. The amount of media exposure was derived from the following questions: (i) Do you read a newspaper or magazine at least once a week, less than once a week, or not at all? (ii) Do you listen to the radio at least once a week, less than once a week, or not at all? (iii) Do you watch television at least once a week, less than once a week, or not at all? Then, the amount of media exposure was calculated by summing up the reported frequency of each media if an activity was performed at least once a week. The household wealth index was constructed using data on a household’s ownership of selected assets, such as televisions and materials used for constructing the house, using the DHS wealth index [41].

### Statistical analyses

Firstly, descriptive analyses were performed and are reported as frequency and percentage with their 95% confidence intervals (CI). To calculate unbiased estimates, the sampling weight, strata, and cluster were incorporated. Secondly, bivariate analyses were conducted using Rao–Scott Chi-square to test the differences between children who did and did not receive DM. Thirdly, multivariate logistic analyses were constructed using generalized linear mixed models with the binomial distribution and the logit link function, since children from the same communities/neighbourhoods/countries may present characteristics that are similar to those of individuals from different communities/countries. Thus, we adjusted for the correlated individual responses nested under a single country using models that handle correlated responses. We reported adjusted odds ratios (aORs) with their *P*-values and 95% CI. We also incorporated survey year fixed effects that control for common time effects across all surveys. We employed multivariable logistic regression to examine the independent factors associated with DM. A significance level of alpha equal to 5% was used to determine the statistical significance. All data entry, cleaning, and statistical analyses were conducted using SAS version 9.4 software (SAS Institute, Cary, NC, USA). We focused on only two levels (i.e. children as level 1 and country as level 2), since we were interested in examining the effects of DM on anaemia among children aged 6–59 months in selected SSA countries; multilevel models with dichotomous outcomes were employed to estimate the odds of success and the impact of various characteristics at different levels.

1 $$Y_{ij} = \beta_{0j} + \beta_{1j} X_{ij} .$$

Equation (1) presented above indicates a simple level 1 model with one child-level predictor, where *Y*_*ij*_ signifies the anaemia status for child *i* in country *j*, *β*_0*j*_ signifies the intercept or the average log odds of the occurrence of anaemia in the country *j*, *X*_*ij*_ is a level 1 predictor for a child *i* in country *j*, and *β*_1*j*_ indicates the slope associated with *X*_*ij*_ showing the link between the child-level variables and the log odds of anaemia occurrence. The regression coefficients are modelled hierarchically as follows:

2 $$\beta_{0j} = \gamma 00 + \gamma 01W_{j} + U_{0j} ,$$

$$\beta_{1j = } \gamma 10.$$

Equation 2 shows a simple level 2 model with one child-level predictor, where *γ*00 provides the log odds of anaemia prevalence among all counties, *W*_*j*_ is a country-level predictor, *γ*01 is the slope associated with this predictor, *U*_0*j*_ is the level 2 error term representing the unique effect associated with country *j*, and *γ*10 is the average effect of the child-level predictor. Therefore, an integration of the level 1 and level 2 models produces the following.

3 $$Y_{ij} = \, \gamma 00 + \gamma 10X_{ij} + \gamma 01W_{j} + U_{0j} .$$

## Results

### Characteristics of the study participants

Table 1 presents the characteristics of the study participants. Overall, a total of 50,075 pre-SAC aged 6–59 months were extracted from 17 SSA countries and analysed in this study. Approximately half of the respondents (49%) were distributed in the age group 25–34 years, and a quarter (25%) had secondary and above education. Additionally, about 2% of the respondents had access to all three forms of mass media (newspaper, radio, and television). With regards to household environmental factors, about two-thirds of respondents (64%) had access to an improved water source, less than half (43%) had improved sanitation facilities, and over three-quarters of the respondents were rural dwellers.

**Table 1:** Characteristics of the study participants in 17 SSA countries, 2014–2019

Variables	Frequency, *n*	Percent, %	(95% confidence interval)
Sex (male)	25,156	50.34	(49.81–50.86)
Age in months			
6–11	5485	10.94	(10.62–11.27)
12–23	11,422	22.83	(22.39–23.27)
24–35	10,971	21.79	(21.36–22.23)
36–47	11,083	22.33	(21.89–22.77)
48–59	11,114	22.10	(21.67–22.54)
Fever in the last 2 weeks	11,650	23.38	(22.94–23.82)
Diarrhoea in the last 2 weeks	7621	15.47	(15.10–15.85)
Child is stunted	18,970	38.37	(37.86–38.88)
Child is wasted	2761	5.28	(5.04–5.52)
Age of the respondents (years)			
< 25	13,264	26.09	(25.63–26.55)
25–34	24,503	49.40	(48.87–49.92)
≥ 35	12,308	24.52	(24.07–24.97)
Education level of the respondents			
No formal education	18,603	38.21	(37.70–38.73)
Primary education	18,525	37.00	(36.50–37.51)
Secondary and above education	12,947	24.78	(24.33–25.23)
Household wealth			
Poorest	13,927	26.26	(25.81–26.71)
Poorer	11,389	23.63	(23.19–24.07)
Middle	10,042	20.60	(20.18–21.02)
Richer	8659	17.85	(17.44–18.26)
Richest	6058	11.66	(11.31–12.01)
Amount of media exposure			
0	30,887	61.78	(61.26–62.28)
1	13,436	26.58	(26.12–27.04)
2	4887	9.82	(9.50–10.13)
3	865	1.82	(1.67–1.97)
Type of drinking water sources (improved)	32,337	63.96	(63.96–64.47)
Type of sanitation facility (improved)	21,772	42.92	(42.40–43.43)
Place of residence (rural)	38,083	77.10	(76.65–77.56)

*SSA* sub-Saharan Africa

### Prevalence of anaemia and other health-related characteristics

**Fig. 3:**
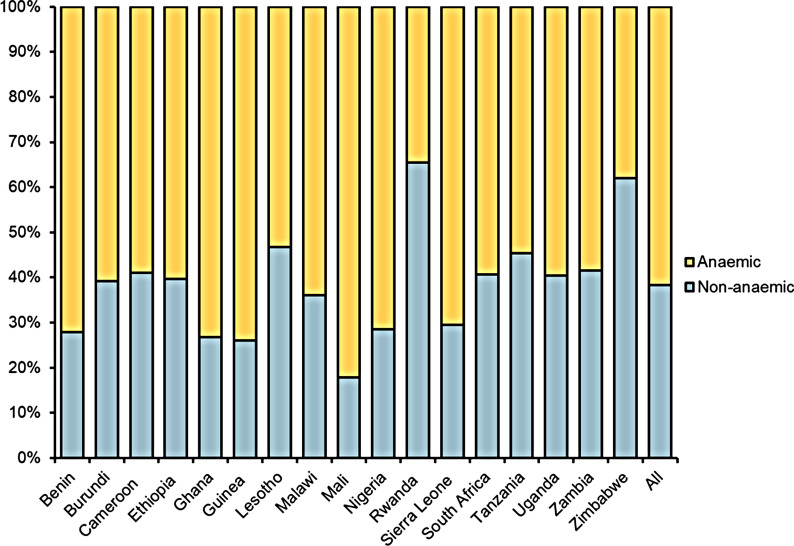
The prevalence of anaemia stratified by countries

**Fig. 4:**
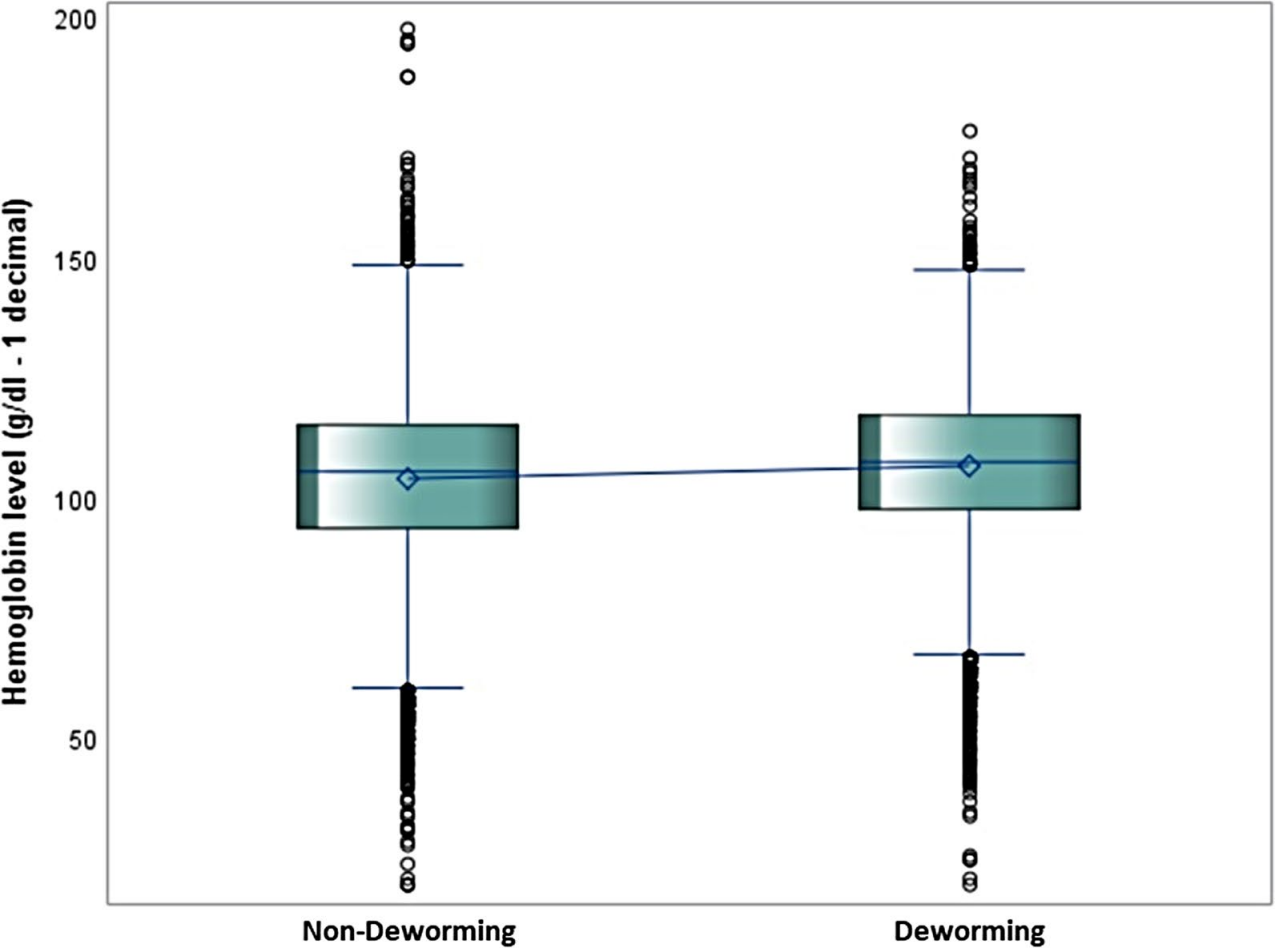
The median haemoglobin concentration among pre-SAC stratified by deworming medicine

**Fig. 5:**
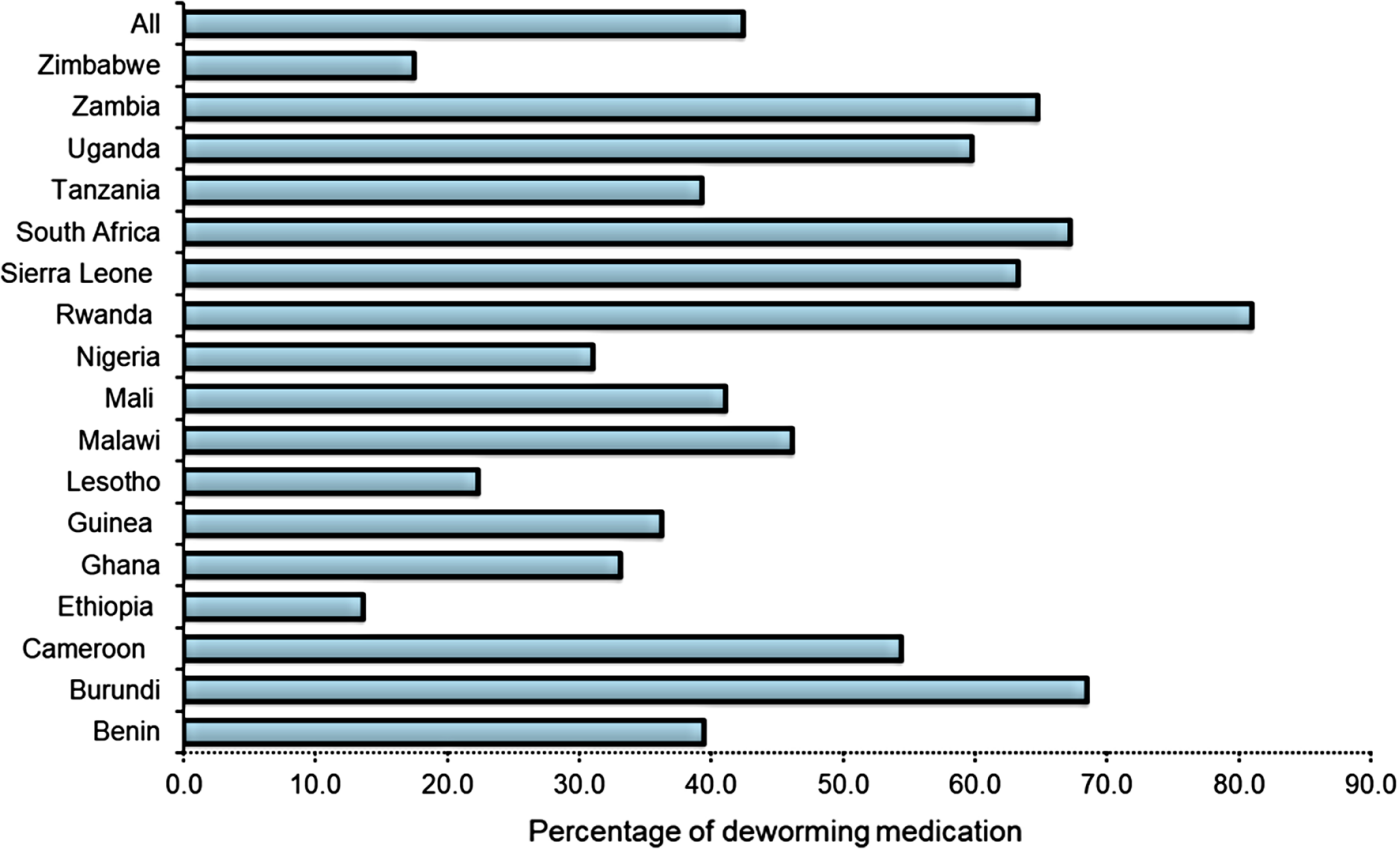
The coverage of deworming medication stratified by countries

Figure 3 shows the results of the prevalence of anaemia in surveyed pre-SAC, where 61.7% (95% CI 61.2–62.2) of the pre-SAC were anaemic. The prevalence of anaemia was highest in Mali (81.1%) and lowest in Rwanda (34.5%). However, the median concentration of Hb was higher among pre-SAC that received DM compared to pre-SAC that did not receive DM, as shown in Fig. 4. Overall, the coverage of DM in SSA was reported at 42.5% (95% CI 41.9–43.0), as presented in Fig. 5. The coverage was highest in Rwanda (81.0%) and lowest in Ethiopia (13.7%). Regarding child health-related outcomes, about 15% and 23% of children had experienced fever and diarrhoea episodes in the prior 2 weeks, respectively. Approximately 38% of children were stunted and 5% of children were wasted.

### Prevalence of anaemia in children aged 6–59 months by selected characteristics

Table 2 presents the prevalence of anaemia among children aged 6–59 months by selected factors. The prevalence was significantly different with respect to DM status (*χ*^2^ = 165.73, df = 1, *P* < .0001), child age (*χ*^2^ = 2138.61, df = 4, *P* < .0001), sex of the child *χ*^2^ = 38.16, df = 1, *P* < .0001), history of reported fever (*χ*^2^ = 409.64, df = 1, *P* < .0001) and diarrhoea (*χ*^2^ = 167.47, df = 1, *P* < .0001) in the last 2 weeks, nutritional status of the child-stunting (*χ*^2^ = 356.99, df = 1, *P* < .0001) and wasting (*χ*^2^ = 118.46, df = 1, *P* < .0001), age of the respondents (*χ*^2^ = 108.83, df = 2, *P* < .0001), education level of the respondents (*χ*^2^ = 916.52, df = 2, *P* < .0001), Hb level of the respondents (*χ*2 = 1629.21, df = 1, *P* < .0001), household wealth (*χ*^2^ = 597.71, df = 4, *P* < .0001), types of water sources (*χ*^2^ = 72.86, df = 1, *P* < .0001), sanitation facilities (*χ*^2^ = 213.69, df = 1, *P* < .0001), place of residence (*χ*^2^ = 115.50, df = 1, *P* < .0001), and year of the survey (*χ*^2^ = 1138.47, df = 5, *P* = 0.001).

**Table 2:** Association of deworming medicine and other risk factors with anaemia in children aged 6–59 months in 17 SSA countries, 2014–2019

Variables	Prevalence of anaemia, % (95% CI)^‡^	Anaemia
		Adjusted odds ratio (95% CI)
Received deworming medicine		
No	36.87 (36.36–33.38)***	1.11 (1.07–1.16)***
Yes	24.80 (24.36–25.25)	1.00
Sex		
Male	31.72 (31.23–32.21)***	1.11 (1.07–1.16)***
Female	29.95 (29.48–30.44)	1.00
Age in months		
6–11	8.52 (8.33–8.81)***	3.86 (3.56–4.18)***
12–23	16.68 (16.28–17.01)	2.83 (2.67–3.01)***
24–35	13.31 (12.95–13.67)	1.60 (1.51–1.70)***
36–47	12.16 (11.82–12.51)	1.23 (1.16–1.30)***
48–59	11.01 (10.68–11.34)	1.00
Fever in the last 2 weeks		
No	7.10 (6.83–7.36)***	0.70 (0.66–0.73)***
Yes	16.28 (15.90–16.66)	1.00
Diarrhoea in the last 2 weeks		
No	4.91 (4.69–5.14)***	0.97 (0.91–1.02)
Yes	10.56 (10.24–10.88)	1.00
Stunting status		
Not stunted	12.71 (12.36–13.06)***	0.74 (0.71–0.77)***
Stunted	25.66 (20.25–26.12)	1.00
Wasting status		
Not wasted	1.49 (1.37–1.62)***	0.86 (0.78–0.94)**
Wasted	3.79 (3.58–3.99)	1.00
Age of the respondents (years)		
< 25	17.06 (16.66–17.45)***	1.14 (1.08–1.20)***
25–34	30.02 (29.54–30.51)	1.11 (1.05–1.15)**
≥ 35	14.59 (14.22–14.96)	1.00
Education level of the respondents		
No formal education	26.74 (26.27–27.21)***	1.53 (1.44–1.62)***
Primary education	21.25 (20.82–21.68)	1.06 (1.00–1.11)*
Secondary and above education	13.67 (13.33–14.04)	1.00
Maternal anaemia		
No	11.14 (10.81–11.47)***	0.51 (0.49–0.53)***
Yes	29.13 (28.65–29.61)	1.00
Household wealth		
Poorest	17.83 (17.44–18.22)***	1.47 (1.35–1.60)***
Poorer	15.10 (14.72–15.47)	1.30 (1.20–1.14)***
Middle	12.51 (12.17–12.86)	1.20 (1.11–1.30)***
Richer	10.31 (9.98–10.65)	1.15 (1.07–1.24)**
Richest	5.92 (5.66–6.17)	1.00
Type of drinking water sources		
Unimproved	25.40 (24.95–25.86)***	0.99 (0.95–1.04)
Improved	38.55 (38.05–39.07)	1.00
Type of household sanitation facility		
Unimproved	36.77 (36.27–37.29)***	0.99 (0.95–1.04)
Improved	24.90 (24.45–25.34)	1.00
Place of residence		
Urban	13.14 (12.77–13.50)***	0.95 (0.90–1.01)
Rural	48.53 (48.01–49.06)	1.00
Year of survey		
2014	2.35 (2.20–2.50)***	0.69 (0.61–0.79)***
2015	9.17 (8.87–9.48)	0.54 (0.49–0.59)***
2016	14.74 (14.34–15.15)	0.65 (0.59–0.71)***
2017	4.40 (4.21–4.60)	0.85 (0.76–0.96)**
2018	27.35 (26.90–27.81)	0.95 (0.87–1.04)
2019	3.64 (3.46–3.83)	1.00

CI: confidence interval; Hb: haemoglobin; g/dl: grams per deciliter

*< 0.05; **< 0.001; ***< 0.0001

^‡^Percentages and *P*-values were derived from Rao–Scott Chi-square test

### The effect of DM on anaemia in children aged 6–59 months

Table 2 also shows the effects of DM on anaemia. Children who did not receive DM were 1.1 times as likely (aOR: 1.11; 95% CI 1.07–1.16; *P* < .0001) to be anaemic as their counterparts.

### Factors associated with coverage of DM among children aged 6–59 months

Table 3 shows factors associated with a recipient of DM among pre-SAC. Children of age group 6–11 months were 77% (aOR: 0.23; 95% CI 0.21–0.24; *P* < .0001) less likely to receive DM compared to children of age group 48–59 months. Compared to children whose respondents were 35 years and above, children whose respondents were 25 years and less were 21% (aOR: 0.79; 95% CI 0.74–0.84; *P* < .0001) less likely to receive DM. Furthermore, children from the poorest households and whose respondents had no formal education were 51% (aOR: 0.49; 95% CI 45–0.53; *P* < .0001) and 52% (aOR: 0.48; 95% CI 45–0.51; *P* < .0001) less likely to receive DM compared to their richest counterparts and whose respondents had secondary education and above, respectively. Additionally, children whose households had no media exposure were 45% (aOR: 0.55; 95% CI 0.48–0.64; *P* < .0001) less likely to receive DM than those from households that had access to all three modes of media (newspaper, radio, and television). Conversely, children from urban areas were 20% (aOR 0.80: 95% CI 0.75–0.85; *P* < .0001) less likely to receive DM compared to their rural counterparts.

**Table 3:** Factors associated with coverage of deworming medicine in children aged 6–59 months, 17 SSA, 2014–2019

Variable	Coverage of DM: % 95% (CI)^‡^	Deworming
		Adjusted odds ratio: 95% (CI)
Sex		
Male	21.40 (20.98–21.82)	1.00 (0.97–1.04)
Female	21.06 (20.64–21.47)	1.00
Age in months		
6–11	2.07 (1.92–2.22)***	0.23 (0.21–0.24)***
12–23	9.45 (9.15–9.75)	0.79 (0.75–0.83)***
24–35	10.35 (10.04–10.66)	1.05 (0.99–1.10)
36–47	10.48 (10.17–10.80)	1.01 (0.96–1.07)
48–59	10.10 (9.80–10.41)	1.00
Age of the respondents (years)		
< 25	10.42 (10.11–10.73)***	0.79 (0.74–0.84)***
25–34	21.13 (20.71–21.55)	0.90 (0.86–0.96)**
≥ 35	10.91 (10.58–11.23)	100
Education level of the respondents		
No formal education	12.59 (12.25–12.93)***	0.48 (0.45–0.51)***
Primary education	17.99 (17.60–18.38)	1.21 (1.14–1.28)***
Secondary and above education	11.88 (11.54–12.22)	1.00
Household wealth		
Poorest	9.37 (9.07–9.66)***	0.49 (0.45–0.53)***
Poorer	9.28 (8.99–9.57)	0.59 (0.54–0.65)***
Middle	8.97 (8.68–9.25)	0.70 (0.64–0.76)***
Richer	8.64 (8.34–8.94)	0.85 (0.79–0.92)***
Richest	6.20 (5.93–6.46)	1.00
Amount of media exposure^†^		
0	23.37 (22.93–23.80)***	0.55 (0.48–0.64)***
1	13.09 (12.75–13.47)	0.76 (0.66–0.88)***
2	4.92 (4.70–15.16)	0.76 (0.65–0.89)***
3	1.07 (0.99–1.19)	1.00
Place of residence		
Urban	11.00 (10.67–11.35)***	0.80 (0.75–0.85)***
Rural	31.44 (30.98–31.92)	1.00
Year of survey		
2014	1.79 (1.65–1.92)***	0.27 (0.24–0.31)***
2015	9.91 (6.65–7.17)	0.22 (0.20–0.24)***
2016	9.45 (9.15–9.76)	0.28 (0.25–0.31)***
2017	3.59 (3.41–3.77)	0.74 (0.65–0.83)***
2018	17.40 (17.00–17.78)	0.37 (0.34–0.41)***
2019	3.32 (3.14–3.50)	1.00

*CI* confidence intervals, *DM* deworming medicine

*< 0.05; **< 0.001; ***< 0.0001

^‡^Percentages and *P*-values were derived from Rao–Scott Chi-square test

^†^Frequency of reading newspaper or magazine, frequency of listening to radio, frequency of watching television

## Discussion

We aimed to examine the effects of DM on anaemia among pre-SAC in selected countries in SSA. The finding on the high prevalence of anaemia is consistent with what was reported by the World Bank [42]. The principal strategies that have been used to control anaemia over the past decades are iron supplementation and food fortification [43, 44]. However, public health programmes that target a large population such as DM have received little attention among pre-SAC. And yet there is empirical evidence on the high prevalence of anaemia attributable to intestinal parasite infection among older children in developing countries.

The most important finding of this study was that children who did not receive DM were more likely to be anaemic. Many previous studies have reported that children who had STHs were more likely to have micronutrient deficiency [45–47]. For example, a study on species-specific STHs and micronutrients (Hb, plasma ferritin, retinol, zinc, and urinary iodine) in Vietnamese SAC found that *Ascaris* infections exhibited a negative association with vitamin A, while *Trichuris* and hookworm infections were associated with lower Hb levels. Furthermore, children who were infected with *Trichuris* were more likely to have zinc deficiency than uninfected children [48].

In Nigeria, a study on STHs and anaemia status of children reported that hookworm and *Ascaris lumbricoides* infections were associated with anaemia. However, it was reported that serum ferritin levels were more sensitive than Hb in detecting anaemia and were correlated with intestinal helminth infection [49]. Thus, STH control in pre-SAC through deworming may contribute to lowering the burden of anaemia as well as increasing Hb levels in pre-SAC. For example, a meta-analysis of eight studies found a significant change in the Hb levels after deworming, and the prevalence of anaemia decreased markedly after the DM programme [29].

It is reported that deworming prevents hookworm infection, which is one of the major causes of iron loss in children, and in turn prevents anaemia [29]. Previously it was suggested that STH infections may cause malabsorption of both macro- and micronutrients. which in turn can interfere with iron metabolism and result in undernutrition, including anaemia, among young children [50]. Typically, *Necator americanus* and *Ancylostoma duodenale* adhere to and feed from the intestinal mucosa, thus causing blood (and iron, protein) loss and, depending on underlying iron status as well as the presence of other risk factors, possibly leading to iron-deficiency anaemia [13]. Furthermore, worms may compete for vitamin A in the intestine and may induce chronic intestinal blood loss that can result in anaemia [14, 15, 51, 52]. Previous researchers reported that low Hb concentration owing to hookworm infection was the largest cause of anaemia in Oceania and East Asia [53]. Elsewhere, hookworm infections were reported to be less prevalent among pre-SAC, and it was suggested that hookworms may not have significant effects on anaemia among this vulnerable group [16]. However, in Malawi, children with hookworm infections were reported to be severely anaemic [22].

We also examined the factors associated with DM coverage and found that young children were less likely to receive DM. To date, no study has ever addressed why young children have a reduced chance of receiving DM. However, many DM programmes in SSA utilize school settings, typically because SAC are thought to be a group with the highest intensity of worm infection [54]. Additionally, it has been proven that school-based targeted deworming programmes provide an opportunity for STH control efforts while utilizing an existing school-based infrastructure to reach a large proportion of SAC, with teachers acting as drug distributors [55]. Moreover, research has demonstrated that when older children are treated with DM, worm infections become less prevalent not only for them, but also for household contacts who reside in the same environment as treated children [56, 57]. Thus, this might explain the small effect size observed between children not receiving deworming treatment being anaemic compared to those who did receive deworming treatment.

We also found that the age of the respondents had a significant impact on the recipient of DM among children aged 6–59 months. Studies on the use of maternal and child health care services in general have reported poor outcomes among young women in SSA [58, 59]. For instance, adolescent girls and young women were less likely to use antenatal care (ANC) than older women [60]. Another study in Nigeria suggested that only a quarter of adolescent women received safe delivery care that was handled by skilled attendants [61, 62]. Additionally, utilization of postnatal care (PNC) was also unsatisfactory, with only a third of adolescent mothers receiving the service in SSA [62]. The reasons leading to such behaviour are often cited as lack of child-bearing experience [63, 64]. Thus, women with such poor health-seeking behaviours may be more likely to default to other important health services such as DM.

We found that children whose respondents had no formal education and resided in the poorest households were less likely to receive DM. The results are consistent with previous studies in resource-limited settings [65–67]. These results imply that women who have better education are more likely to comprehend the new knowledge and are more likely to know where to get care for their children. Additionally, education may empower women to have greater confidence, change of poor attitude, and capability of making a decision to use modern health services for themselves and for their children, including DM [68]. Previous studies have reported that higher income may be associated with a higher prospect to acquire improved health knowledge and health-seeking behaviour [69, 70], including DM. More importantly, wealthier households might be more likely to purchase or obtain DM [71].

We used data from samples that were nationally representative in all the included countries; therefore, our results may be generalized to the selected countries that were included in this study. The analysis controlled for a wide range of covariates, thereby strengthening the observed associations. We are aware of the potential limitations of the study. The findings cannot strictly be treated as causal due to the cross-sectional nature of the study designs. Given the nature of the variables, there is a potential for identification problems which may result in endogeneity [72]. Therefore, future research should aim at adopting quasi-experimental methods to address this issue [73].

## Conclusions

The current study revealed empirical evidence of increased anaemia among children who had not received DM. Thus, targeted public health strategies that are aimed at reducing anaemia in pre-SAC should also consider population-based interventions such as DM. Further, in order to increase the uptake and coverage of DM, which eventually may improve Hb concentration of pre-SAC, socio-economically disadvantaged women should be targeted. However, longitudinal research is needed in order to validate the association that has been revealed in this current study. These results have policy implications in the sense that the WHO focus on DM could be extended to pre-SAC, a group most likely to be missed by interventions.

## Data Availability

The datasets generated and/or analysed during the present study are available in the DHS Program repository. https://dhsprogram.com/data/dataset/Malawi_Standard-DHS_2015.cfm?flag=1

## Abbreviations

ANC: Antenatal care
aOR: Adjusted odds ratio
CDC: Centers for Disease Control and Prevention
CI: Confidence intervals
DHSs: Demographic and health surveys
DM: Deworming medication
Hb: Haemoglobin
IRB: Institutional review board
JMP: Joint Monitoring Programme
NTDs: Neglected tropical diseases
Pre-SAC: Preschool-age children
SAC: School-age children
SSA: Sub-Saharan Africa
STH: Soil-transmitted helminth
UNICEF: United Nations Children’s Fund
WHO: World Health Organization
